# The Role of the PAX Genes in Renal Cell Carcinoma

**DOI:** 10.3390/ijms25126730

**Published:** 2024-06-19

**Authors:** Lei Li, Sultana Mehbuba Hossain, Michael R. Eccles

**Affiliations:** 1 Department of Pathology, Dunedin School of Medicine, University of Otago, Dunedin 9016, New Zealand; lile2757@student.otago.ac.nz (L.L.); mehbuba.hossain@otago.ac.nz (S.M.H.); 2 Maurice Wilkins Centre for Molecular Biodiscovery, Level 2, 3A Symonds Street, Auckland 1010, New Zealand

**Keywords:** renal cell carcinoma, subtypes, *PAX* genes, *VHL*, clear cell renal cell carcinoma

## Abstract

Renal cell carcinoma (RCC) is a significant oncological challenge due to its heterogeneous nature and limited treatment options. The *PAX* developmental gene family encodes nine highly conserved transcription factors that play crucial roles in embryonic development and organogenesis, which have been implicated in the occurrence and development of RCC. This review explores the molecular landscape of RCC, with a specific focus on the role of the *PAX* gene family in RCC tumorigenesis and disease progression. Of the various RCC subtypes, clear cell renal cell carcinoma (ccRCC) is the most prevalent, characterized by the loss of the von Hippel–Lindau (*VHL*) tumor suppressor gene. Here, we review the published literature on the expression patterns and functional implications of *PAX* genes, particularly *PAX2* and *PAX8*, in the three most common RCC subtypes, including ccRCC, papillary RCC (PRCC), and chromophobe RCC (ChRCC). Further, we review the interactions and potential biological mechanisms involving *PAX* genes and *VHL* loss in driving the pathogenesis of RCC, including the key signaling pathways mediated by *VHL* in ccRCC and associated mechanisms implicating *PAX*. Lastly, concurrent with our update regarding *PAX* gene research in RCC, we review and comment on the targeting of *PAX* towards the development of novel RCC therapies.

## 1. Introduction

Renal cell carcinoma (RCC) is the predominant form of kidney cancer, representing approximately 80–90% of kidney cancers, and it primarily originates from the renal tubular epithelium or renal cortex [1,2]. According to the 2020 Globocan Registry, RCC ranks as the 12th most common cancer globally, with 1.2 million cases reported over the past five years [3]. In 2020, the global age-standardized incidence rate (ASR) for RCC was 4.6 per 100,000 individuals. The ASR for men (6.1 per 100,000) was nearly double that for women (3.2 per 100,000) [3]. The incidence of RCC varies significantly by region, with higher rates generally observed in Europe and North America [4]. The gradual increase in the risk of RCC over the past decade is noteworthy and warrants greater attention.

With its diverse histological and molecular characteristics, RCC is classified into three main subtypes: clear cell renal cell carcinoma (ccRCC), papillary renal cell carcinoma (PRCC), and chromophobe renal cell carcinoma (ChRCC). Among these, ccRCC is the most prevalent, accounting for approximately 70–90% of cases, followed by PRCC with 10–15% of cases, and ChRCC with 3–5% of cases [5,6]. Additionally, rare types of RCC account for less than 1% of incidence each. Approximately 90% of sporadic ccRCC cases exhibit an alteration of the Von Hippel–Lindau tumor-suppressor gene (*VHL*), located on chromosome 3 [7].

Normal kidney development is a highly orchestrated process involving a multitude of genes that regulate the rapid proliferation and differentiation of embryonic tissues. PAX proteins are vital for organ development, but their expression typically diminishes post-development [8]. The *PAX* genes (*PAX1–PAX9*) regulate cell processes, including proliferation, differentiation, apoptosis, migration, and stem cell maintenance [8,9]. Dysregulation of the *PAX* genes has been linked to a variety of diseases, including cancer [8,9]. *PAX2* and *PAX8* are frequently expressed during kidney development, whereas other *PAX* genes either show low expression or are not expressed [10,11]. As the nascent nephron matures, *PAX2* expression is downregulated in the pedunculated progenitor cells of the developing glomerulus and subsequently in all proximal and distal tubules of the nephron [12,13]. *PAX8* is still persistently expressed in epithelial cells of fully developed nephrons [14]. However, it is noteworthy that *PAX2* was found to be reactivated in renal cell carcinoma and that around 90% of renal cell carcinoma patients have high *PAX2* and *PAX8* expression levels [5,15]. 

*PAX* genes are thought to originate during evolution from a proto-*PAX* ancestor. The highly conserved PD (N-terminal paired domain) motif, which contains 128 amino acids, is present in all PAX proteins [16]. The genetic and functional homology observed in *PAX* genes across species highlights their evolutionary stability, suggesting strong evolutionary pressure to preserve these motifs due to their critical roles in development and cellular function. The *PAX* gene family is categorized into subgroups I–IV based on the presence or absence of an octapeptide region and the presence, absence, or truncation of a homeodomain. Of these, both the PD and homeodomain (HD) motifs show DNA binding ability [16], although only a partial HD is observed in the *PAX2* and *PAX8* genes. The DNA binding motifs enable PAX proteins to function as transcription factors and play crucial roles in embryonic development. During embryogenesis, mutations in subgroup I members (*PAX1* or *PAX9*) have been associated with congenital vertebral malformations, Klippel–Feil syndrome, and oligodontia [17,18]. Mutations in the *PAX2* gene cause coloboma syndrome [19]. Interestingly, *PAX2* is not normally expressed in healthy adult kidneys, although it becomes reactivated in RCC [20,21]. In our previous investigations, we found that the knockdown of *PAX2* sensitized RCC cells to cisplatin-induced apoptosis [22]. *PAX3* has been associated with the survival of melanoma cells and is considered essential for the viability of melanoma cell lines [23]. *PAX4* predominantly exhibits expression in the pancreas during embryonic development, and mutations in the *PAX4* gene have been implicated in the development of diabetes mellitus [24]. *PAX5* serves as a key driver in B-cell malignancies [25]. Mutations in *PAX6* lead to conditions like “small eye” in mice and aniridia, congenital cataracts, or Peter’s anomaly in humans [26]. *PAX8* was found to be frequently expressed in RCC patients, and validation from a large-scale functional genomic screen confirmed that *PAX8* silencing leads to reduced proliferation of RCC cell lines [27]. Moreover, disturbances in *PAX* function are linked to cancerous growth in humans, such as rhabdomyosarcoma (involving *PAX3* and *PAX7*), non-Hodgkin lymphoma (involving *PAX5*), follicular thyroid carcinoma (involving *PAX8*), and RCC (involving *PAX2* and *PAX8*) further implicating the functional significance of *PAX* genes (Table 1) [28,29,30].

Several members of the *PAX* gene family have been found to be expressed in RCC, and their expression was found to be diagnostic and potentially a therapeutic target for RCC [20,27,31]. In this review, we discuss the expression characteristics and potential roles of *PAX* genes in RCC. In addition, we discuss the molecular characteristics of different RCC subtypes, especially regarding the identified relationship between *VHL* mutations and the potential role of *PAX* genes in RCC.

## 2. Recruitment of *PAX* Gene Expression Enables Renal Cell Carcinomas to Survive, Proliferate, and Metastasize

**Figure 1:**
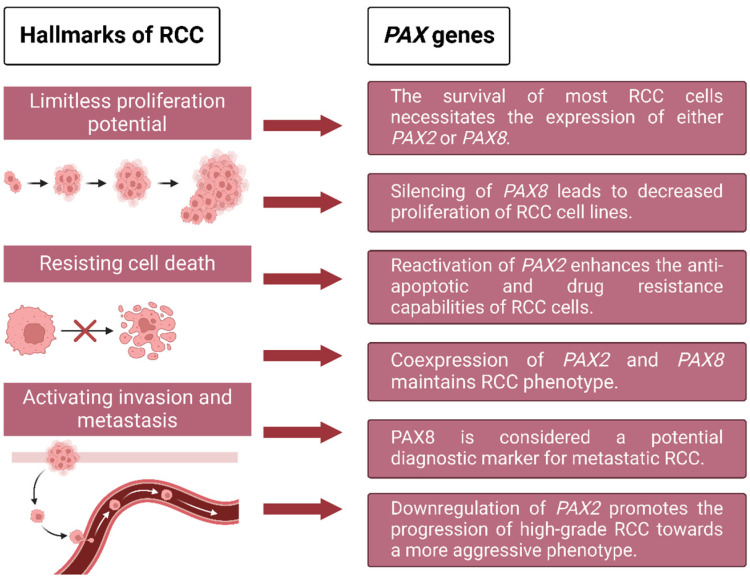
The hallmarks of RCC: exploring the potential role of *PAX* genes. *PAX* genes primarily participate in three hallmarks of RCC: cell survival, proliferation, and metastasis. Among these hallmarks, *PAX2* and *PAX8* are implicated in the transformation of RCC phenotype, either individually or cooperatively.

The *PAX* family of proteins comprises developmental regulatory proteins crucial for normal tissue development [8,9]. These proteins possess DNA binding domains that encode partially or fully functional transcription factor-associated domains to regulate the expression of a range of downstream genes [9,32]. *PAX* gene expression undergoes tight regulation during development. *PAX2* and *PAX8* are expressed during the early stages of urogenital development [33,34], playing a crucial role in the differentiation of renal precursor cells in the intermediate mesoderm and the transformation of metanephric mesenchymal cells into epithelial cells within developing kidney nephrons [34,35]. In general, *PAX* genes are not expressed in adult proximal and distal tubules. However, the *PAX2* gene is selectively re-expressed to modulate and enhance kidney regeneration, such as after renal tissue injury [36]. While *PAX* genes are transiently expressed during kidney development, their deregulation has been associated with certain kidney cell abnormalities in humans [8,35]. The recruitment of *PAX* gene expression, especially *PAX2* and *PAX8*, plays an important regulatory role in the survival, proliferation, and metastasis of RCC (Figure 1).

### 2.1. PAX Genes in Renal Cell Carcinoma Survival and Proliferation

Cancer entails a multi-step process characterized by cumulative alterations in both positive and negative regulators of cell proliferation and survival. The frequent expression of *PAX* genes in cancer is deemed essential for cancer cell growth and survival [33]. Distinct survival dependencies on *PAX2* are observed in ovarian and bladder cancer cell lines [33]. Additionally, overexpression of *PAX2* in Kaposi’s sarcoma cells was found to enhance their resistance to apoptotic signals [37]. Other PAX genes are also involved in cell survival. In melanoma cell lines, for example, the inhibition of *PAX3* induces apoptosis and markedly impedes cell survival [33]. Additionally, *PAX4* functions as a survival gene in INS-1E insulinoma cells [38]. Notably, *PAX8* is recognized as the prototypical lineage survival oncogene in epithelial ovarian cancer, and dysregulation of *PAX8* promotes the progression of ovarian cancer [39].

In RCC, *PAX2* and *PAX8* exhibit heightened expression levels compared to other *PAX* genes [5]. *PAX2* is among the earliest genes expressed in the kidney lineage and plays a crucial role in the survival, proliferation, and functional differentiation of kidney lineage cells [19]. Reactivation of *PAX2* expression in RCC correlates with the anti-apoptotic capacity of RCC cells, while its inhibition helps to promote RCC cell apoptosis and sensitization to chemotherapy drugs such as cisplatin [22,31]. During epithelial-to-mesenchyme (EMT) transition of RCC cells, silencing of *PAX2* expression reduces *ADAM10* expression, which induces L1-CAM-dependent activation of the PI3K/Akt pathway, which is crucial for RCC cell survival [40].

Similarly, *PAX8* plays a pivotal role in the survival and proliferation of epithelial cells [41]. The silencing of *PAX8* leads to decreased proliferation in RCC cell lines [27], inducing apoptosis through a p53-dependent pathway, which involves caspase-3 activation and poly(ADP)ribose polymerase cleavage [41]. Notably, within the metabolic dysregulation hallmark of renal cell carcinoma, *PAX8* is implicated in activating metabolic genes via enhancer elements [27]. Interaction of the PAX8 protein with hypoxia inducible factor 2 subunit alpha (HIF2α) recruits the latter to transcriptional enhancers, promoting downstream oncogenic signaling pathways and supporting the expression of typical oncogenes like *CCND1* and *MYC* [42,43].

Moreover, *PAX2*, *PAX8*, and the paralogous *PAX5* have been demonstrated to inhibit tumor suppressor gene *TP53* expression by directly binding to its first exon, thereby suppressing apoptosis and promoting tumor progression [44]. This inappropriate expression of *PAX* genes not only impedes *TP53* tumor suppressor function but also activates specific target genes that facilitate tumorigenesis [44,45].

### 2.2. PAX Genes in Renal Cell Carcinoma Metastasis

EMT involves a cellular mechanism wherein epithelial cells undergo a phenotypic change, adopting a mesenchymal phenotype characterized by enhanced motility and invasiveness [46]. Local and distant dissemination constitutes one of the hallmarks of tumor progression, with EMT significantly contributing to the metastatic potential of tumors. The expression profile and biological properties of EMT underscore its potential as a therapeutic target in cancer, including RCC. In RCC, EMT signifies a transient shift of tumor cells from an epithelial to a mesenchymal state, facilitating tumor cell motility, invasion, and metastasis in advanced stages. Conversely, mesenchymal–epithelial transition (MET) halts cell migration and promotes colonization in distant organs [46]. Loss of, or suppression of E-cadherin expression emerges as a pivotal event in EMT, associated with heightened expression of transcriptional repressors such as ZEB1, ZEB2, Slug, Snail, and Twist [47], some of which are known to repress E-cadherin expression. Additionally, EMT has been associated with an elevated risk of recurrence and poorer overall survival (OS) in RCC patients [47]. Targeting dysregulatory factors that drive EMT may improve the prognosis of patients with RCC.

The expression of both *PAX2* and *PAX8* is positive in most patients with metastatic RCC [20]. The reactivation of *PAX2* in the early stages of RCC is fundamental to the development of the RCC phenotype. *PAX2* is involved in a complex interplay with *ADAM10* and the TGF-β1 signaling pathway, exerting regulatory control over RCC progression and metastasis [40,48]. ADAM10, which is a member of a family of transmembrane and secreted proteins, plays an important role in the progression and metastasis of various cancers [40]. PAX2 binds to the promoter of the *ADAM10* gene in RCC and regulates ADAM10 protein expression in RCC cells [40]. Moreover, silencing *PAX2* expression results in decreased *ADAM10* expression, leading to a more scattered cell phenotype accompanied by induction of *SNAI2* (Slug) expression and loss of *CDH1* expression (E-cadherin), which is observed during EMT. Furthermore, downregulation of *ADAM10* reduces RCC cell proliferation, while *PAX2* knockdown results in increased L1 cell adhesion molecule (L1CAM) expression, which then induces endothelial cell proliferation and RCC cell migration. Some studies have noted a decline in *PAX2* expression levels in high-grade RCC [49,50]. It is thought this reduction in *PAX2* expression plays a contributory role in driving RCC towards a more aggressive phenotype.

PAX8 is recognized as a promising diagnostic marker for metastatic RCC, demonstrating higher sensitivity compared to PAX2 [20]. Several groups have identified key target genes regulated by PAX8, among them being GATA binding protein 3 (GATA3), LIM homeobox protein 1 (LHX1), and Wilms tumor 1 (WT1) transcription factor [51,52]. These transcription factors, along with PAX2 and PAX8, collectively form a core transcription factor circuit that plays a crucial role in driving proximal tubular epithelial fates. PAX8 has been implicated in regulating the tumor microenvironment by enhancing fibronectin and collagen expression while increasing TGF-β1 secretion [53]. *PBRM1* deletion was found to be the second most prevalent genetic alteration in RCC [54], and loss of *PBRM1* coactivator in RCCs is thought to lead to an imbalance in coregulator content within a PAX8-associated master transcription factor hub for the kidney lineage. This imbalance favors the over-representation of co-repressors, resulting in the repression of the terminal proximal tubule epithelial program [55].

## 3. Different Renal Cell Carcinoma Subtypes Are Distinguished by Molecular Features with Various Roles

Determining the source/origin of metastatic cancer is important, as it greatly influences the choice of local treatment strategy and systemic therapy. Different levels of *PAX* gene expression are observed in different RCC subtypes. Gupta et al. conducted a study involving 200 renal tumors and found that almost all ccRCC were immunoreactive for PAX2 (28/30, 93%), whereas more than half (53%) of PRCC expressed PAX2, while most of ChRCC (94%) were non-immunoreactive for PAX2 [15]. We previously also found high expression of *PAX2* and *PAX8* mRNA in ccRCC and PRCC cell lines, whereas these genes were expressed at low levels or not at all in ChRCC cell lines [5,32].

### 3.1. Molecular Features of Different Renal Cell Carcinoma Subtypes

Classical PRCC is often associated with concurrent gains of chromosomes 7 and 17, alongside infrequent gains of chromosomes 2, 3, 12, 16, or 20 [56,57]. Chromosome 7 contains several potential oncogenes, including *MET*, *EGFR*, and *BRAF* [57]. Somatic activating mutations of *MET* have been identified in a subset of classical PRCC cases, while hereditary PRCC stems from germline activating mutations of *MET* [57]. An analysis conducted by TCGA revealed somatic or germline *MET* mutations in 18.6% of classical PRCC cases, accompanied by occurrences of somatically acquired alternative *MET* RNA transcripts and *MET* gene fusions [58]. These alterations, along with the frequent gain of chromosome 7, were found to collectively contribute to *MET* modifications in classical PRCC. Genetic studies on metastatic ChRCC have revealed high-risk features associated with unfavorable survival outcomes, such as *TP53* mutations, *PTEN* mutations, DNA hypermethylation, and imbalanced chromosome duplication [59]. Most ChRCCs exhibit complete losses of several chromosomes (1, 2, 6, 10, 13, 17, and 21) [60,61].

ccRCC is characterized by a near-total deletion of the short arm of chromosome 3, resulting in the loss of multiple tumor suppressor genes [62]. Additionally, numerous genomic alterations have been identified in ccRCC, primarily involving epigenetic reprogramming and modifications in oncogenic metabolism pathways [63]. Frequently mutated genes include those regulating the cellular oxygen pathway (e.g., *VHL*) and maintaining chromatin structure integrity (e.g., *PBRM1* and *BAP1*) [64,65]. *BAP1* and *PBRM1* are situated near the *VHL* region on chromosome 3p [66,67]. The VHL protein (pVHL) is a crucial component of the oxygen-sensing system responsible for hypoxia inducible factor (HIF) degradation. Mutations or loss of function in the *VHL* gene result in the stabilization of HIF-1/2-α subunits, activating HIF target genes [68,69]. This pathway is implicated in various processes such as tumor angiogenesis, invasion, cancer metabolic reprogramming, and metastasis. Although *VHL* inactivation is nearly ubiquitous in ccRCC, it alone is insufficient to cause the disease. Preneoplastic cysts have been observed with *VHL* loss, and even mice with *VHL* disruption in the kidneys do not develop ccRCC, even when both alleles of *VHL* are inactivated in the appropriate kidney compartment [70,71]. These findings suggest that ccRCC tumorigenesis requires additional driver mutations to cooperate.

### 3.2. The Role of VHL Loss in Clear Cell Renal Cell Carcinoma

**Figure 2:**
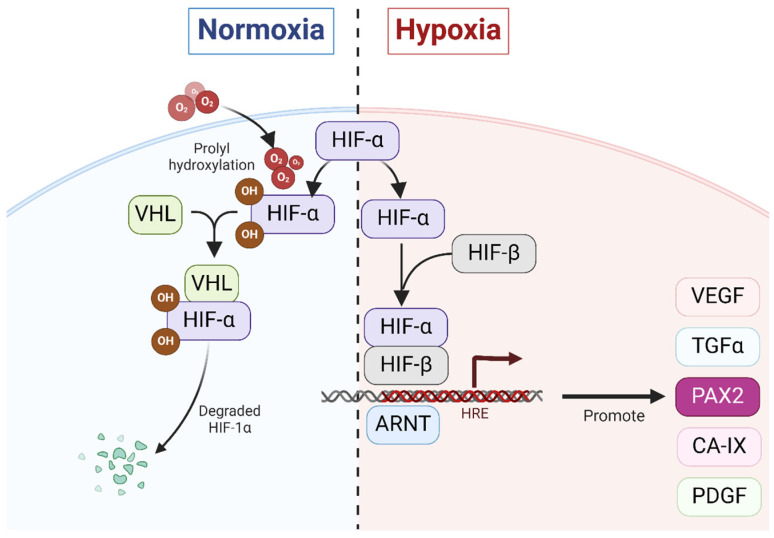
*VHL* signaling participates in the regulation of cellular adaptation to hypoxia and contributes to reactivated *PAX2* expression in ccRCC. During normoxic conditions, HIF-α undergoes hydroxylation by prolyl hydroxylases (PHD) and subsequently becomes recognized by the VHL protein. Upon binding of VHL to HIF-α, the latter undergoes ubiquitination and subsequent degradation through the proteasome machinery. Conversely, under hypoxic conditions, HIF-α remains un-hydroxylated, leading to its accumulation within the cytosol. This accumulated HIF-α then translocates into the nucleus, where it forms a heterodimer with its counterpart, HIF-β. The resultant complex drives the transcription of genes possessing a hypoxia-responsive element (HRE) within their promoters and further promotes the expression of their potential target genes (including *VEGF*, *PDGF*, *CA-IX*, *TGFα*, and *PAX2*). Furthermore, it is noteworthy that in ccRCC, *PAX2* reactivation is driven by HIF-dependent mechanisms following pVHL loss [36].

*VHL* functions as a subunit in a larger protein complex that recognizes specific protein substrates, initiating their ubiquitination and subsequent degradation by the proteasome [72,73]. This enables *VHL* to regulate cellular responses to fluctuating oxygen levels, ensuring appropriate cellular adaptations to hypoxic environments (Figure 2). Loss of *VHL* can disrupt hypoxia signaling pathways in ccRCC, impairing HIF regulation and leading to HIFα accumulation and constitutive expression of HIF target genes. Elevated HIFα levels then interact with hypoxia-responsive components, resulting in abnormal upregulation of genes like *VEGF*, platelet-derived growth factor (*PDGF*), *CA-IX*, *TGFα*, and *PAX2* which contribute to ccRCC development [74,75].

Following *VHL* loss, overexpression of HIF-2α is necessary and sufficient for tumor growth [76]. In addition, *VHL* deficiency can lead to mitochondrial dysfunction [77]. Perrotta et al. found that *VHL* defects affect energy metabolism and impair mitochondrial function [77]. Mitochondrial dysfunction encompasses structural damage, defects in the respiratory chain, genetic abnormalities, reduced mitochondrial number, and altered oxidative protein activity [78]. The kidney is one of the body’s highest energy-demanding organs, second only to the heart in oxygen consumption and mitochondrial abundance [79]. Aerobic respiration is the primary mechanism for production of adenosine triphosphate (ATP) [80]. Renal tubular epithelial cells are highly dependent on ATP production via oxidative phosphorylation (OXPHOS) for epithelial transport functions [81]. The persistence of mitochondrial dysfunction underscores the protraction of tubular injury, thereby potentially impeding the process of renal recuperation following an episode of acute kidney injury (AKI). Moreover, this unrelenting dysfunction serves as a catalyst for the advancement of chronic kidney disease (CKD) [82].

Mitochondrial dysfunction occupies a pivotal role in the developmental trajectory of RCC [42,83]. Most cancer cells, instead of relying on OXPHOS for ATP production even when sufficient oxygen is available, primarily derive ATP from glycolysis [84]. This phenomenon, termed “aerobic glycolysis”, was identified by Warburg many years ago [85]. In ccRCC, this shift to aerobic glycolysis is attributed to hypoxia-induced stabilization of the transcription factor HIF, which is further facilitated by *VHL* inactivation [86]. Mitochondrial transcription factor A (*TFAM*) plays a crucial role in activating mitochondrial transcription and replication [87]. EGLN3 hydroxylates TFAM, and hydroxylated TFAM then binds to pVHL. This binding with pVHL stabilizes TFAM, protecting it from mitochondrial protein hydrolysis [87]. Furthermore, a deficiency of *TFAM* was shown to trigger mitochondrial dysfunction in HK2 (Human Kidney-2) cells [87]. Metabolic perturbations are characteristic of many tumor types, especially RCC [88]. Mitochondrial dysfunction engenders augmented reliance on substrate-level phosphorylation for energy production [89]. These *VHL* tumors have been found to display reduced mitochondrial content, indicating that dysfunctional mitochondrial biogenesis might be linked to *VHL* tumorigenesis.

## 4. Signaling Pathways with Important Roles in Renal Cell Carcinoma and Their Relationship to *PAX*

**Figure 3:**
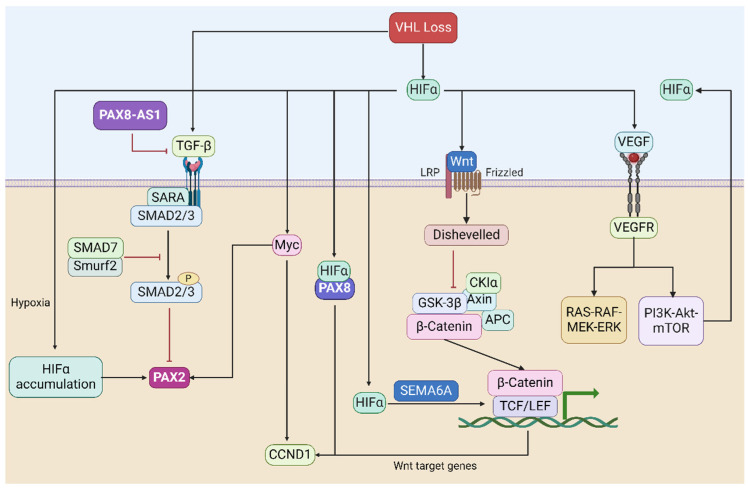
*VHL*-mediated signaling/regulatory pathways in ccRCC with respect to *PAX* involvement. The loss of *VHL* leads to HIFα accumulation and translocation into the nucleus, which subsequently activates the transcription of HIF target genes that are involved in critical oncogenic pathways (such as HIF-based hypoxia signaling pathway, TGF-β signaling pathway, VEGF-related signaling pathways, MYC-related signaling pathways, and Wnt/β-catenin signal pathway). Loss of *VHL* promotes the progression and metastasis of RCC, wherein *PAX* genes also exert a positive contribution.

The occurrence and progression of cancer are frequently linked to the dysregulation of signaling pathways. Within RCC, a diverse range of histological subtypes exists, each characterized not only by distinct molecular signatures but also increasingly by unique molecular pathways that undergo deregulation in each subtype [90]. Loss of *VHL* triggers the accumulation and nuclear translocation of HIFα, subsequently activating crucial oncogenic signaling pathways in RCC. The absence of *VHL* seems to serve as an early foundation for RCC development, with the *PAX* genes also contributing and being upregulated by the *VHL*-mediated hypoxia signaling pathway (Figure 3).

### 4.1. The HIF-Based Hypoxia Signaling Pathway

Hypoxia (a state of oxygen deficiency) is a prominent characteristic within numerous solid malignancies, primarily attributed to inadequate perfusion and/or the development of poorly or non-functional blood vessels within neoplasms. This phenomenon is particularly pronounced in rapidly proliferating tumors. In most ccRCC, hypoxia-inducible factors (HIFs), which are global transcriptional regulators of the hypoxic response, are persistently activated through the inactivation of a component of the E3 ubiquitin ligase complex, the VHL tumor-suppressor protein [91].

The *VHL* tumor-suppressor gene mediates and participates in the transduction of the hypoxia signaling pathway through HIFα [91] (see Figure 3). Luu et al. showed that *PAX2* reactivation in ccRCC is induced by hypoxia and depends on the functional integrity of pVHL and that *PAX2* reactivation after pVHL loss is driven by HIF-dependent mechanisms [92]. HIF is a heterodimer complex comprising a labile α subunit and a stable β subunit [93]. The α subunit is the major regulatory subunit of HIF. Its level is mainly regulated by intracellular oxygen concentration. Under normoxic conditions, hydroxylation of the HIFα subunit enables its recognition and ubiquitination by the pVHL complex [94], thereby keeping intracellular HIFα levels low. Conversely, during hypoxia, HIFα remains unhydroxylated, resulting in its non-interaction with VHL proteins and consequent lack of degradation. Isono et al. found that HIF2α is overexpressed in VHL-deficient RCC cells, leading to higher cell viability in tumor cells [95]. Additionally, in a ccRCC xenograft model, HIF-2α overexpression in RCC cells contributes to increased tumor burden [76]. When HIF-1α was overexpressed, a reduction in tumor size was observed, whereas the knockdown of HIF-1α increased RCC cell proliferation [76]. Therefore, HIF-1α may exert a tumor suppressor function in the context of RCC.

### 4.2. The TGF-β Signaling Pathway

In RCC, the TGF-β pathway is often dysregulated, leading to tumor promotion [96]. TGF-β acts as a tumor suppressor in the early stages of RCC, inhibiting cell proliferation and promoting differentiation. However, in later stages, TGF-β may promote tumor progression by inducing EMT. TGF-β1 can exert its functions through both canonical (Smad-dependent) and non-canonical (Smad-independent) signaling pathways [97]. Canonical TGF-β1 signaling (Smad-based) can promote RCC progression by promoting invasion and metastasis and inducing EMT [98]. TGF-β signaling has been found to interact with HIF to promote renal fibrosis [99]. Inhibition of *PAX2* transcription in ccRCC cells through classical TGF-β signaling has been reported [48] (see Figure 3). *LncRNA-PAX8-AS1* was found to be located in chromosome 2q13 upstream of the *PAX8* gene and has a negative regulatory effect on *PAX8* [100]. *PAX8-AS1* can bind to *miR-96-5p* to inhibit the malignant phenotype of papillary thyroid carcinoma cells [101]. In renal peduncle cells, *PAX8-AS1* reduces renal peduncle apoptosis and inhibits the expression of *VEGF*/*TGF-β1* [102]. Shang et al. showed that *TGF-β1* may be a direct target of *VHL* rather than regulating *TGF-β1* expression through the HIF pathway [103]. Furthermore, the inactivation of *VHL* in ccRCC may enhance the metastatic ability of RCC cells by increasing *TGF-β1* expression [103].

### 4.3. The VEGF-Related Signaling Pathway

*VEGF* is a cytokine that plays a central role in promoting the formation of angiogenesis [104]. In cancer, increased *VEGF* expression and angiogenesis are crucial for tumor growth and metastasis [104]. *VEGF* was found to be a potential HIFα target in ccRCC, and knockdown of HIFα decreased the expression level of *VEGF* [105]. Elevated *VEGF* expression is frequently linked to poor prognosis in RCC patients [106]. VEGF, upon binding to its corresponding cell surface receptors such as VEGFR, induces tyrosine phosphorylation of the receptor, initiating downstream signaling through various kinase-dependent pathways, including the PI3K-Akt-mTOR pathway and the RAS-RAF-MEK-ERK pathway [107,108] (see Figure 3). PAX2 has been reported to be related to angiogenesis in RCC [109]. Research by Fonsato et al. shows that PAX2 may participate in the Akt pathway and affect angiogenesis by regulating PTEN [109]. PTEN is a phosphatase that regulates PI3K-dependent activation of Akt. PAX8 was found to promote angiogenesis in ovarian cancer by interacting with SOX17, but whether it is related to angiogenesis in RCC remains unclear [110]. It is important to highlight that mTOR is among the downstream effectors of PI3K/AKT, and its activation results in increased expression levels of HIFα [107]. Notably, an aberrant *VHL* function can establish a harmful positive feedback loop. The absence of *VHL* leads to impaired degradation of HIFα, causing its accumulation at high levels. Subsequently, elevated HIFα levels further stimulate the abnormal expression of VEGF, which binds to VEGFR, activating the PI3K-Akt pathway. Consequently, mTOR is also activated through this pathway, leading to even higher HIFα levels. This vicious positive feedback loop exacerbates the defects caused by aberrant *VHL* function, thereby promoting the onset and progression of ccRCC.

### 4.4. The MYC-Related Signaling Pathway

*MYC* is a family of regulatory and proto-oncogenes encoding transcription factors (-*MYC* (MYC), *L-MYC* (MYCL), and *N-MYC* (MYCN)) [111,112]. *MYC* regulates various biological processes, including cell differentiation, cell growth, apoptosis, and stem cell self-renewal [111,112]. Hwang et al. showed that pVHL can bind to the *C-MYC* promoter along with C-MYC and then transcriptionally repress the *C-MYC* gene [113]. In metanephric mesenchyme cells, N-MYC acting downstream of PAX2 may restore *PAX2* expression at the transcriptional level and thus regulate cell proliferation and apoptosis [114]. HIF-2α can promote *C-MYC* activity and induce cell cycle progression in ccRCC cells [115]. On the other hand, HIF-1α acts as an inhibitor of C-MYC activity, leading to reduced levels of E2F and cyclin D2 while concurrently enhancing the expression of cell cycle progression inhibitors p21 and p27 [115,116]. Another significant discovery is that HIF-1 inhibits mitochondrial biogenesis and cellular respiration in *VHL*-deficient renal cell carcinoma by impeding *C-MYC* activity [117]. C-MYC can transactivate *CCND1* expression [118] (see Figure 3). *CCND1* plays a tumor-promoting role in RCC, and its knockdown has been shown to attenuate xenograft growth in vivo [119]. Additionally, *CCND1* is considered a potential downstream gene of HIF-2α. Its expression decreases upon HIF-2α inhibition and increases upon re-expression of HIF-2α in vitro [76]. Notably, HIF-2α and PAX8 interact with each other at the level of chromatin, and they may be synergistically involved in the regulation of *CCND1* expression [120].

### 4.5. The Wnt/β-Catenin Signaling Pathway

Wnts represent a family of secreted glycoproteins responsible for regulating essential cellular processes, including cell proliferation, differentiation, and migration [121]. Multiple lines of evidence suggest the involvement of the Wnt/β-catenin signaling pathway in RCC [122,123] (see Figure 3). Blockade of the WNT signaling pathway inhibits the migration and invasion ability of PAX2-positive cells [124]. Wnt1 was found to be significantly overexpressed in ccRCC, and high levels of Wnt1 are associated with more aggressive RCC cells and worse prognosis in patients [123]. Among the intracellular signal transducers, β-catenin plays a crucial role as a participant in the canonical Wnt signaling pathway [125,126]. Unphosphorylated β-catenin can interact with members of the LEF and TCF families, thereby further promoting the transcription of Wnt target genes (such as *C-MYC* and *CCND1*) [127,128]. While β-catenin-activating point mutations are not common in RCC, studies have demonstrated that inducing overexpression of β-catenin leads to the development of renal tumors in mice [129]. Ji et al. showed that activation of the Wnt/β-catenin pathway correlates with the expression of SEMA6A in ccRCC [122]. The VHL-HIF-2α axis can stabilize β-catenin and promote ccRCC progression by inducing SEMA6A upregulation [122].

## 5. The Development of *PAX*-Related Therapeutic Strategies in Renal Cell Carcinoma

In recent years, advancements in medicine have continuously updated and optimized the treatment of RCC. Surgery remains the primary treatment for RCC, especially for localized and locally advanced renal cancer. However, the emergence of immune checkpoint inhibitors (ICIs) has dramatically transformed the treatment prospects for RCC. Currently, the standard first-line treatment for advanced ccRCC includes ICI combinations. The dual immune checkpoint blockade with ipilimumab and nivolumab, targeting cytotoxic T lymphocyte-associated protein 4 (CTLA-4) and programmed cell death protein 1 (PD-1), respectively, is the first ICI combination approved by the US Food and Drug Administration (FDA) under the CheckMate 214 trial [130,131]. ICI combination therapy has brought overall survival benefits and improved prognostic characteristics to some patients. Nevertheless, a significant number of patients exhibit primary resistance to ICI combinations or develop acquired resistance, ultimately succumbing to the disease [131]. Therefore, the development of new treatment options and the identification of novel therapeutic targets to enhance the prognosis of RCC remain the primary focus of current RCC research.

Genetic loss-of-function studies suggest that *PAX* genes, particularly *PAX2* and *PAX8*, which are highly expressed in RCC, are crucial for the continued proliferation and/or survival of RCC cells. Consequently, targeting *PAX2* or *PAX8* may represent a potentially effective therapeutic approach for the treatment of RCC (Table 2). Previous studies have utilized small interfering RNA (siRNA) to knock down *PAX2*, demonstrating that *PAX2* knockdown sensitized RCC cells to cisplatin-induced apoptosis, resulting in the death of 50–60% of cisplatin-resistant ACHN and CAKI-1 cells [22]. Further animal experiments have corroborated these findings, indicating that silencing *PAX2* expression could partially overcome the resistance of RCC to chemotherapy in vivo [31]. Additionally, large-scale functional genomic screening validated that *PAX8* silencing led to reduced proliferation of RCC cell lines [27]. Grimley et al. have screened large chemical libraries and identified a compound, EG1, that can effectively block PAX2 activity and DNA binding through the PAX2 paired domain [132]. Their findings revealed that EG1 can significantly inhibit the proliferation of *PAX2*-positive RCC cells while having minimal impact on *PAX2*-negative RCC cells. Recently, Bradford et al. [133] discovered three triazolo pyrimidine derivatives that inhibit *PAX*-mediated reporter gene transcriptional activation. Analysis of cultured renal epithelial cells demonstrated that these small molecules specifically inhibit PAX2 and PAX8 function by preventing the recruitment of the adaptor protein PTIP and the assembly of histone methylation complexes at target promoters. Furthermore, these compounds can specifically slow the proliferation of *PAX2*-positive RCC cells [133]. Combining *PAX* targeting with immunotherapy may represent a promising therapeutic direction. However, progress in developing therapeutic strategies targeting PAX proteins remains inadequate, particularly regarding the development of *PAX*-related inhibitors, which face significant obstacles. One major challenge arises from the inherently disordered structure of PAX proteins, rendering direct targeting approaches complex. Additionally, the predominant nuclear localization of PAX proteins poses significant hurdles to effective drug delivery. Addressing these challenges demands concerted efforts to explore novel methodologies and deepen our understanding of *PAX* genes, thereby facilitating the development of more efficacious therapeutic interventions.

## 6. Conclusions

The *PAX* gene family plays an important role in the occurrence and development of RCC [8,9]. *PAX2* undergoes reactivation during the early stages of ccRCC [12,13]. Approximately 90% of RCC tumors exhibit high *PAX2* and *PAX8* expression levels, while other *PAX* genes have low or no expression in RCC [5,15]. Moreover, it is noteworthy that *PAX2* and *PAX8* exhibit heightened expression frequencies in both ccRCC and PRCC, while their expression remains either low or entirely absent in ChRCC. The upregulation of *PAX2* and *PAX8* within the context of RCC may be intricately linked to the maintenance of the epithelial phenotype during the process of EMT. Several lines of evidence implicate *PAX2* and *PAX8* as promising, emerging potential therapeutic targets for the treatment of RCC.

The most frequently mutated gene in ccRCC is *VHL*, where approximately 90% of sporadic ccRCC cases show alterations in the *VHL* gene located on chromosome 3 [7]. Notably, the loss of *VHL* function results in the upregulation of *PAX2* expression in ccRCC. Loss of *VHL* alone is insufficient to induce ccRCC, which requires additional epigenetic events to occur. When the *VHL* gene mutates or loses its function, the HIF-1/2-α subunit becomes stable, leading to the activation of *HIF* target genes and their downstream signaling pathways [72,73,92]. *HIF-2α* is preferentially recruited to *PAX8*-bound transcriptional enhancers, which together coordinate the expression of the oncogene *CCND1* [120]. In conclusion, the loss of *VHL* in ccRCC may be involved in the occurrence and development of ccRCC in conjunction with *PAX* gene expression through *HIF*-mediated downstream target genes and signaling pathways.

Targeting *PAX2* or *PAX8* may be a potentially valuable treatment approach for RCC [12,31]. Small molecule compounds that target *PAX* have been developed and employed in targeted cancer therapy (non-clinical), which yielded promising in vitro results [132,133]. However, it is important to note that the expression levels of *PAX2* undergo dynamic changes during RCC development, reflecting a phenotypic shift in *RCC*. *PAX2* is reactivated in early ccRCC, but its expression levels begin to decline in high-grade ccRCC. This coincides with ccRCC transitioning towards a more aggressive phenotype and favoring glycolysis for energy production. Consequently, in patients with high-grade ccRCC, regardless of whether *PAX2* is targeted or not, its expression has decreased. The potential reasons for this phenomenon could include metabolic reprogramming of RCC and alterations in the tumor microenvironment, although the specific mechanisms remain unclear. Therefore, the clinical application of *PAX* gene inhibitors will require a further understanding of the role of *PAX* genes in cancer.

The generation of small molecule compounds that directly target PAX proteins is at a relatively early stage in terms of *PAX* inhibitor drug development because in vivo delivery of *PAX*-targeting small molecules is a significant barrier. Similarly, in vivo stability and delivery of siRNAs against *PAX* mRNAs are also significant barriers. Consequently, there remain significant gaps in the development of targeted therapies for *PAX* in RCC. Additionally, there continues to be ongoing debate regarding the use of *PAX2* expression levels as a metastatic marker for RCC. One potential reason for this debate may be the discrepancies in methods used to quantify gene expression, whether through the detection of RNA or protein markers. Although mRNA levels are early indicators of gene expression, they do not necessarily correlate directly with protein levels due to the influence of various regulatory factors such as translation efficiency and protein degradation rates. Furthermore, the expression of *PAX* genes varies in different regions of RCC at different times.

Future investigations could prioritize the elucidation of the potential mechanisms associated with *PAX* involvement in RCC and advancing therapeutic strategies targeting *PAX*. Presently, the precise mechanisms underlying *PAX*-mediated RCC onset and progression remain elusive. Notably, *PAX* genes are frequently expressed during developmental processes and are implicated in tissue regeneration and repair. Emerging research suggests a correlation between *PAX* expression and signaling pathways associated with stemness, indicating that the reactivation of *PAX* genes in RCC may contribute to the manifestation of cancer stemness characteristics [134,135]. Moreover, combination therapies targeting multiple pathways hold promise as a burgeoning approach. Identified potential targets upregulated in RCC, such as MUC1, AXL, EGFR, C-MET, PD-L1, VEGF, HIF, etc., could be targeted in combination with targeting *PAX*, offering valuable support in the development of potential therapeutic strategies for RCC [134,135,136,137,138].

## Figures and Tables

**Table 1:** *PAX* family members.

Groups	* *PAX* Genes	HL	Structural	EA	ER	RC	References
I	*PAX1*	20p11	PD+OCT	Skeleton, thymus	No or low expression	Netural or favourable	[8,17,18]
	*PAX9*	14q12	PD+OCT	Skeleton, craniofacial, tooth	No or low expression	Netural or favourable	[12,17,18]
II	*PAX2*	10q24	PD+OCT+partial HD	CNS, kidney, eye, ear	High expression	Tumor-promoting	[19,20,21,22]
	*PAX5*	9p13	PD+OCT+partial HD	CNS, B cells, testis	No or low expression	Netural or favourable	[8,12,25]
	*PAX8*	2q12	PD+OCT+partial HD	CNS, kidney, thyroid	High expression	Tumor-promoting	[8,10,27]
III	*PAX3*	2q35	PD+OCT+HD	CNS, neural crest, skeletal muscle	No or low expression	Netural or favourable	[8,12,23]
	*PAX7*	1p36	PD+OCT+HD	CNS, craniofacial, skeletal muscle	No or low expression	Netural or favourable	[8,12]
IV	*PAX4*	7q32	PD+HD	CNS, Pancreas	No or low expression	Netural or favourable	[8,12,24]
	*PAX6*	11p13	PD+HD	CNS, eye, pancreas	No or low expression	Netural or favourable	[8,26]

* The *PAX* family may be categorized into four subgroups (Groups I–IV) based on the distinctive assembly of three structural motifs: the amino (N)-terminal paired domain (PD), the octapeptide (OCT), and the homeodomain (HD). Both the PD and HD motifs demonstrate DNA binding capabilities. The highly conserved PD motif comprises 128 amino acids and is ubiquitous among all PAX proteins. Group I (*PAX1* and *PAX9*) entirely lack HD, while group IV members (*PAX4* and *PAX6*) have a complete HD but lack an OCT domain. Group III members (*PAX3* and *PAX7*) feature a complete HD and an OCT domain, whereas Group II members (*PAX2*, *PAX5*, and *PAX8*) possess a partial HD. HL: human chromosome location; EA: expression area; CNS: central nervous system; ER: expression levels in RCC; RC: RCC contribution.

**Table 2:** Strategies used for *PAX*-related therapeutic approaches in RCC.

*PAX*	Methods	Model	Main Outcomes	Authors, Year [Reference]
*PAX2*	SiRNA inactivation of PAX2	RCC cell lines	PAX2 inactivation enhances cisplatin-induced apoptosis in renal carcinoma cells	Hueber et al., 2006 [22]
*PAX2*	shRNA targeting PAX2	RCC cell lines and nude mice	Subcutaneous ACHN/shPAX2 xenografts in nude mice respond better to cisplatin therapy than control ACHN tumors	Hueber et al., 2008 [31]
*PAX2*	Virtual screening and experimental validation	RCC cell lines	A small molecule inhibitor (EG1) targeting the DNA binding domain of PAX2	Grimley et al., 2017 [132]
*PAX8*	Large-scale functional genomic screens (ChIP-seq, RNA-seq, and ATAC-seq)	RCC cell lines	PAX8 silencing results in decreased proliferation of RCC cell lines	Bleu et al., 2019 [27]
*PAX2*	Unbiased cell-based high-throughput screening assay	RCC cell lines	Three triazolo pyrimidine derivatives were identified that inhibited PAX-mediated transcriptional activation of reporter genes and suppressed proliferation of PAX2-positive RCC cells.	Bradford et al., 2022 [133]
